# Tools and Approaches for Dissecting Protein Bacteriocin Import in Gram-Negative Bacteria

**DOI:** 10.3389/fmicb.2019.00646

**Published:** 2019-03-28

**Authors:** Iva Atanaskovic, Colin Kleanthous

**Affiliations:** Department of Biochemistry, University of Oxford, Oxford, United Kingdom

**Keywords:** bacteriocin, import, Gram-negative bacteria, cell envelope, methods

## Abstract

Bacteriocins of Gram-negative bacteria are typically multi-domain proteins that target and kill bacteria of the same or closely related species. There is increasing interest in protein bacteriocin import; from a fundamental perspective to understand how folded proteins are imported into bacteria and from an applications perspective as species-specific antibiotics to combat multidrug resistant bacteria. In order to translocate across the cell envelope and cause cell death, protein bacteriocins hijack nutrient uptake pathways. Their import is energized by parasitizing intermembrane protein complexes coupled to the proton motive force, which delivers a toxic domain into the cell. A plethora of genetic, structural, biochemical, and biophysical methods have been applied to find cell envelope components involved in bacteriocin import since their discovery almost a century ago. Here, we review the various approaches that now exist for investigating how protein bacteriocins translocate into Gram-negative bacteria and highlight areas of research that will need methodological innovations to fully understand this process. We also highlight recent studies demonstrating how bacteriocins can be used to probe organization and architecture of the Gram-negative cell envelope itself.

## Introduction

**FIGURE 1:**
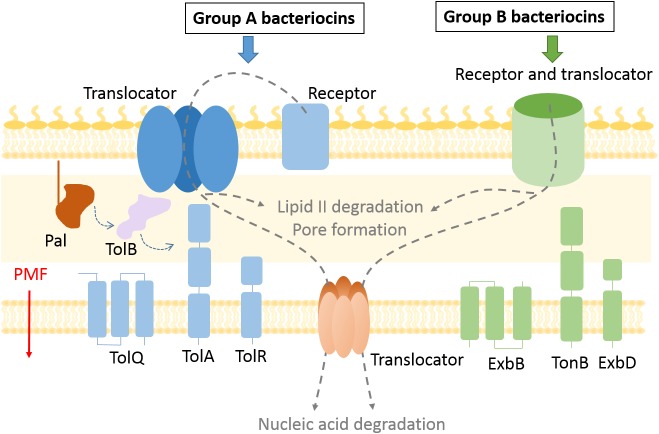
Bacteriocin import pathways. Bacteriocins bind to outer membrane receptors to get imported into the cell. Some bacteriocins (group B) use the receptor protein also as a translocator to cross the outer membrane, while exploiting the TonB system and PMF as an energy source. In case of group A bacteriocins the translocator differs from the receptor protein and the Tol system is used to enter the periplasm. Bacteriocins that degrade lipid II and prevent peptidoglycan recycling remain in the periplasm, while pore forming bacteriocins are inserted in the inner membrane. Nuclease bacteriocins use a distinct protein translocator to cross the inner membrane.

When exposed to environmental or competition stress bacteria often release proteinaceous toxins called bacteriocins that target and kill neighboring bacteria (Cascales et al., 2007; Cornforth and Foster, 2013). Bacteriocins of Gram-positive bacteria are mostly post-translationally modified peptides with a broad species target range, the best known example being the widely used food preservative nisin. Peptidic bacteriocins have been reviewed extensively (Héchard and Sahl, 2002; Cotter, 2014). The focus of the present review is on protein bacteriocins from Gram-negative bacteria. These are large folded proteins (40–80 kDa) that are composed of multiple domains and tend to have a narrow killing spectrum because of the numerous specific interactions at the cell surface involved in their import. A bacteriocin producer cell is protected from its own bacteriocin by an immunity protein, while sensitive strains lack such protection. These toxins can form pores in the inner membrane, act as nucleases to degrade DNA or RNA in the target cell, or interfere with cell wall biosynthesis. To do so, protein bacteriocins (hereafter referred to merely as bacteriocins) have to cross a multi-layered cell envelope which is accomplished by parasitizing host proteins involved in nutrient and metabolite trafficking (Figure 1).

Bacteriocins have the potential to be developed as much-needed therapeutics to treat multidrug resistant bacterial infections (Cotter, 2014; Behrens et al., 2017). A prerequisite for successfully applying bacteriocins as antimicrobials, however, is to understand how they are imported. Moreover, these import pathways might reveal further processes that could be exploited for newly designed drugs or chimeric bacteriocins with more potent toxicity (Lukacik et al., 2012).

The discovery of new bacteriocins has been accelerated by whole-genome sequencing technologies and implementation of gene mining tools (Jamet and Nassif, 2015; Sharp et al., 2017). While peptide Gram-negative bacteriocins, such as microcins, have been reviewed elsewhere (Duquesne et al., 2007), here we focus on methods used for the study of multidomain protein bacteriocins. The two most explored groups of protein bacteriocins are colicins produced by *Escherichia coli* (Cascales et al., 2007) and pyocins produced by *Pseudomonas aeruginosa* (Ghequire and De Mot, 2014). Nuclease colicins contain an N-terminal translocation domain, a central receptor binding domain, and a C-terminal cytotoxic domain that binds a cognate immunity protein, while in pyocins the location of the translocation and receptor binding domains appears reversed (Michel-Briand and Baysse, 2002). Bacteriocins can be divided into two groups based on the periplasmic energy transducing system they exploit for import. Group A use the Tol–Pal system, which is composed of periplasmic and IM proteins, Pal, TolA, TolB, TolR, TolQ. All but the outer membrane lipoprotein Pal have been documented to be involved in bacteriocin import. Group B use the Ton system, composed of TonB, ExbB, and ExbD proteins (Figure 1). It is likely that all protein bacteriocins fall into these two such groups (Kleanthous, 2010).

Colicin E9 is one of the best understood of the Group A bacteriocins in terms of its translocation mechanism. Import of ColE9 involves assembly of a translocon complex. The OM portion of the translocon includes BtuB, its receptor, its porin translocator OmpF or OmpC, TolB, its periplasmic target, and Im9, its immunity protein. In order to form this OM translocon, ColE9 uses its intrinsically unstructured N-terminus to pass through a porin channel to engage the PMF-coupled Tol–Pal system in the periplasm (Housden et al., 2005). How ColE9 translocates across the OM is not understood, but it is known that the colicin exploits FtsH, the AAA^+^ ATPase/protease, to cross the IM. Once inside the cell, the ColE9 DNase causes nonspecific cleavage of double-stranded DNA which results in cell death (Walker et al., 2007). Group B colicins and pyocins pass through Ton-dependent receptors without the involvement of porins. For instance, pyocin S2 binds to a TonB-coupled siderophore receptor FpvAI, and passes through the FpvAI lumen mimicking its cognate ligand (White et al., 2017).

**FIGURE 2:**
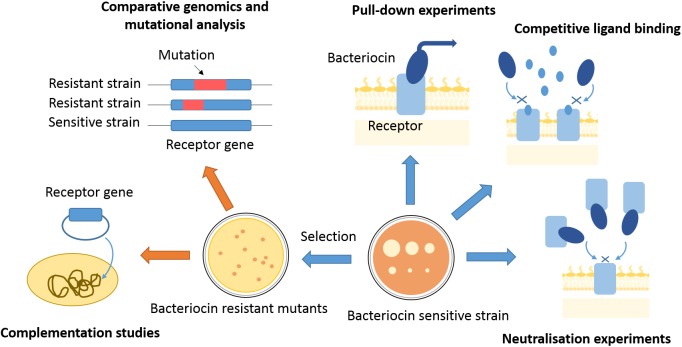
Approaches to finding bacteriocin translocon components. Translocon components can be isolated from membranes of sensitive strains by use of affinity tagged bacteriocins as bait. Competitive ligand binding or bacteriocin binding to a purified translocon component can inhibit bacteriocin activity and give indications about proteins engaged in bacteriocin entry. Bacteriocin-resistant mutants can be selected by use of high bacteriocin concentrations, or generated by transposon mutagenesis. Genomes of resistant mutants can further be analyzed for mutations underlying the resistance phenotype, which can give gene candidates for translocon components. A component of bacteriocin entry has to be further confirmed by complementation studies, where bacteriocin sensitivity is re-established once a resistant mutant is transformed with a gene of interest.

Gram-negative bacteriocins use a variety of pathways and distinct combinations of cell envelope proteins to kill cells, making it challenging to dissect their import mechanisms. Since their discovery, genetic, structural, biochemical, and biophysical approaches have all been deployed to define these pathways often in combination (Figure 2). Here, we give an overview of these approaches, pointing out the most recent advancements in the toolkit used for dissecting bacteriocin translocation in Gram-negative bacteria.

## Pairing Up Bacteriocins With Their Receptors

Every bacteriocin begins its journey through the cell envelope by binding to a specific receptor on the bacterial surface. While most bacteriocins bind OM proteins, some use LPS as their primary receptor (Kim et al., 2014; McCaughey et al., 2014, 2016). The specificity of a bacteriocin–receptor interaction narrows down the target range of these toxins. Therefore, pairing up bacteriocins with their receptors is an important first step in applying them as therapeutics (Cotter, 2014). If a sufficient number of bacteriocin receptors are known, a screen for receptor genes in a genome of a pathogen isolated from the site of infection could guide the design of bacteriocin cocktails to specifically eradicate the cause of infection. Additionally, linking a bacteriocin to a receptor of known biological function is the starting point for understanding which import pathway is being hijacked by the toxin.

When searching for a bacteriocin receptor, the first issue to address is its chemical nature. Bacteriocin *neutralization experiments* have often been used to determine if the receptor is within the protein or LPS fraction of the outer membrane (Weltzien and Jesaitis, 1971). If a certain cell fraction contains the receptor, it will bind to a bacteriocin and inhibit its toxic activity, which can be assessed by a plate killing assay (Figure 2). Neutralization experiments can also be used to test if a specific nutrient import pathway is being hijacked by a bacteriocin. If cells are exposed to a bacteriocin in the presence of a nutrient with which it shares its import pathway, competitive binding of the ligand should either inhibit bacteriocin activity or nutrient import. Such experiments were successful in early attempts at receptor discovery. For instance, competitive binding of cyanocobalamin and E-type colicins gave early indications that they all share BtuB as a receptor (Masi et al., 1973). Similarly, such experiments suggested that pyocin S3 and pyoverdine both bind to the ferripyoverdine type II receptor in *P. aeruginosa* (Baysse et al., 1999), and ferredoxin and pectocin M bind to the same ferredoxin receptor in *Pectobacterium artrosepticum* (Grinter et al., 2012).

An alternative approach is one that combines *neutralization assays with cell wall fractionation and protein purification*. OM fractions can be tested for bacteriocin neutralization activity. After singling out the OM fraction with neutralization activity, further analysis by mass spectrometry can identify the receptor. Use of protease inhibitors is important for preventing OM proteases from degrading the bacteriocin, which can be misinterpreted as bacteriocin neutralization. Misinterpretations can also be avoided by co-fractionating OM proteins isolated from a bacteriocin resistant and a bacteriocin sensitive strain, where all OM proteins from each strain have been labeled with two different fluorescent or radioactive labels. Since the resistant mutants should lack the receptor, a fraction with receptor activity should contain only proteins that originate from the sensitive strain (Sabet and Schnaitman, 1973). A limitation of this approach is that receptor concentrations obtained by OM fractionation can often be insufficient to achieve neutralization. This approach is therefore limited to cases where bacteriocin receptors are expressed to high levels or bind the bacteriocin with high affinity.

Instead of testing for neutralization activity, bacteriocin OM receptors can be identified directly by *pull-down experiments*, using a bacteriocin of interest as a bait (Figure 2). A bacteriocin should co-elute from the chromatography column with its receptor, where further analysis of the co-eluent by proteolysis and peptide fingerprinting by mass spectrometry can give a receptor candidate. For example, Housden et al. (2005) used a hexahistidine-tagged immunity protein complexed to ColE9 and immobilized on a nickel affinity column to purify components of the ColE9 translocon from OM extracts. These pull-down experiments can be challenging in cases of low-abundance receptors. One way to circumvent this problem is to find receptor overproducers, marked by an increased sensitivity to a bacteriocin of interest (Bowles et al., 1983). Another option is to cultivate cells in conditions that increase bacteriocin sensitivity by inducing receptor expression (Bindereif et al., 1982). Defining growth conditions where a bacteriocin receptor is overexpressed can also give valuable hints about the nature of the receptor. This was the case for several S-type pyocin receptors where it was observed that killing of *P. aeruginosa* was more effective if cultivated in iron-limited conditions (Smith et al., 1992; Sano et al., 1993). This observation indicated that siderophore receptors, which are overexpressed when cells are starved of iron, are involved in import of S-type pyocins. Receptors for pyocins S3, S2, S4, and S5 (Baysse et al., 1999; Denayer et al., 2007; Elfarash et al., 2012, 2014) were all discovered by this route.

**FIGURE 3:**
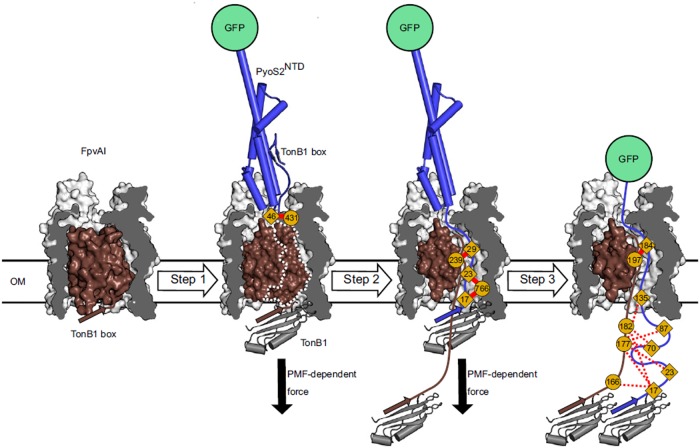
*In vivo* cross-linking strategy developed by White et al. for dissecting the import pathway of pyocin S2 through the FpvAI receptor. Photoactivatable crosslinking data were used to establish a three-step model of translocation. In step 1, pyocin S2 binds to FpvAI and mimics its cognate ligand, ferro-pyoverdine. In step 2, a PMF-dependent mechanical force, applied via the ExbB–ExbD–TonB1 complex in the IM (not shown), drives unfolding of the labile half of the receptor plug domain and the N terminus of pyocin S2 enters the receptor lumen. In step 3, pyocin S2 binds to TonB1 and this interaction drives further passage of the pyocin through the receptor lumen. Complete translocation is blocked by the force-resistant GFP, which enables identification of translocation intermediates and mapping of interactions that govern pyocin S2 cell entry [image taken and used with permission from White et al. (2017), CC BY-NC-ND 4.0].

Another approach for receptor discovery is based on the isolation and characterization of *bacteriocin-resistant mutants*. A resistant mutant can lack a bacteriocin receptor; hence, picking up genetic differences between resistant and sensitive strains can pinpoint the receptor gene (Figure 2). When selecting for resistant mutants by use of lethal bacteriocin doses a major challenge is to distinguish between resistant and tolerant strains; only resistant strains bare mutations that specifically alter import machinery components. Therefore, it is always necessary to disregard tolerance by exposure to even higher bacteriocin doses than those used for selection and by checking if a mutant’s growth kinetics is affected by the bacteriocin. Finally, resistance can be confirmed by checking if the mutant’s OM has lost its bacteriocin binding properties by a neutralization assay or use of a fluorescently labeled bacteriocin to test for cell surface association (Figure 3; Rassam et al., 2015; White et al., 2017).

After isolating a resistant mutant, it is possible to compare its OM protein composition with the parental strain. A combination of electrophoresis and mass spectrometry can point out proteins absent in the OM of the resistant strain, but again, this has only been successful in cases where receptor genes are highly expressed (Ohkawa et al., 1980; Baysse et al., 1999). The advent of *whole-genome sequencing and comparative genomics* is now the preferred method of choice. Comparing a mutant’s genome with a bacteriocin-sensitive reference strain can pinpoint potential receptor genes (McCaughey et al., 2014). This strategy has been successful in identifying bacteriocin receptors in Gram-positive bacteria (Cotter, 2014). The decreasing cost of DNA sequencing and continuous refinement of bioinformatics tools makes it likely that researchers in the field of Gram-negative bacteriocin import will increasingly turn to whole-genome sequencing. However, comparative genomics still has its limitations especially in the case of bacterial strains with a high rate of spontaneous mutations, where it can be difficult to filter out a mutation associated with bacteriocin resistance.

Receptor genes have also been identified by use of *cosmid libraries*. Genomic fragments from a bacteriocin-sensitive strain can be transformed into a bacteriocin-resistant background. The goal is to identify a fragment that can restore bacteriocin sensitivity and potentially carries a receptor gene (Pilsl et al., 1999; Smajs and Weinstock, 2001). An alternative approach is to construct *a library of transposon mutants* in a strain that is sensitive to the bacteriocin. Sequencing of genomic regions around the transposon insertion site in a resistant mutant can pinpoint genes linked to bacteriocin import (Baysse et al., 1999; de Chial et al., 2003; Elfarash et al., 2014; Ghequire et al., 2017). The power of this approach can be increased with the implementation of high-throughput transposon insertion sequencing techniques, such as TraDIS (Barquist et al., 2016), since the employment of a dense transposon library can give better genomic coverage and increase the probability of a resistance phenotype being detected. TraDIS has recently been used to show that LPSs bearing O-antigens shield bacteriocin receptors in uropathogenic *E. coli* but that this effect is modulated by growth conditions (Sharp et al., 2019). Nevertheless, if a receptor is an essential gene no transposon mutants will be obtained and a library search will fail to show the receptor. In this case, pull-down experiments or linkage analysis (see below) can be used.

Bacteriocin receptors can also be found by *linkage analysis*, as in the case of S-type pyocin receptors. S pyocins kill *P. aeruginosa* better under iron limiting conditions, which gave indications that their activity might be linked to the pyoverdine import system (Ohkawa et al., 1980; Smith et al., 1992). A collection of *P. aeruginosa* strains was screened for pyocin S2 sensitivity and ferripyoverdine receptor genes typed by multiplex PCR. All S2 sensitive strains had the type I ferripyoverdine *fpvA*I gene, indicating this was the S2 receptor (Denayer et al., 2007). The same approach was used to link pyocin S4 sensitivity to the *fpvAI* receptor gene (Elfarash et al., 2012). Another type of linkage analysis that was successful in bacteriocin receptor discovery was *metabolite analysis*, where pyoverdine production was compared between pyocin S3 resistant and sensitive strains. It was found that pyocin S3 kills only type II pyoverdine producers of *P. aeruginosa*, while type I and III producers were resistant to S3 (Govan, 1978; Baysse et al., 1999). This gave strong indications that pyocin S3 binds to the ferripyoverdine type II receptor, which was confirmed by subsequent studies (de Chial et al., 2003). A future challenge will be implementation of high-throughput linkage approaches, which can be used when there are no initial clues about the import mechanism. This could be achieved through *genome wide association studies*, if both a genome sequence database and a physical strain collection are available. One could then test bacteriocin sensitivity throughout the collection and conduct a gene linkage analysis for a collection of corresponding annotated genomic sequences (Brynildsrud et al., 2016). Genes that are present in a large number of sensitive, but absent in a large number of bacteriocin resistant strains, are then further tested for receptor coding activity. On the other hand, the development of new mass spectrometric approaches in high-throughput metabolomics (Zampieri et al., 2017) could enable full metabolome comparison between bacteriocin resistant and sensitive strains, where metabolites lacking in resistant strains could give hints about import mechanisms being hijacked by the bacteriocin.

The majority of bacteriocin receptors identified to-date are proteins; however, non-proteinaceous receptors have also been identified. Pyocin L1 is a lectin-like bacteriocin produced by *P. aeruginosa*. It consists of tandem MMBL domains and kills cells by targeting the CPA of *P. aeruginosa* LPS, which is predominantly a homopolymer of D-rhamnose. The widespread inclusion of D-rhamnose in the LPS of pseudomonads explains the unusual genus-specific activity of this lectin-like bacteriocin. The discovery of the pyocin L1 saccharide receptor was achieved through a combination of genetics, structural, and biophysical approaches (McCaughey et al., 2014). Alignment of the pyocin L1 protein sequence with other lectin-like bacteriocins revealed the presence of three conserved MMBL sugar-binding domains, giving the first indications that pyocin L1 might bind to polysaccharide rather than to a protein receptor. A *P. aeruginosa* strain sensitive to this pyocin was used to recover resistant mutants, the genome sequences of which showed a deletion in the *wbpZ* gene, which encodes a glycosyltransferase involved in the synthesis of the CPA component of LPS (Rocchetta et al., 1998). Subsequent immunoblotting with a CPA-specific antibody along with transposon insertions in genes *wzt* and *wzm*, which encode the ATP-binding and the membrane components of a CPA dedicated ABC transporter (Lam et al., 2011), confirmed that CPA on the cell surface is required for pyocin L1 killing. Direct binding of pyocin L1 to CPA and D-rhamnose was shown by ITC and NMR spectroscopy. Finally, X-ray crystallography defined the mode of binding of the D-rhamnose receptor to the C-terminal MMBL domain of pyocin L1. This study therefore provides an excellent example how a combination of genomics and mutational analysis combined with biophysics and structural data can identify non-proteinaceous bacteriocin receptors.

In summary, bacteriocin receptors come in many different types and so an equally varied toolkit is needed for their identification (Figure 2). Receptors with high expression levels can be pulled-down using a bacteriocin as bait. This might be the only viable approach if the receptor is essential. If non-essential, receptor coding genes can be discovered through isolation of bacteriocin-resistant mutants, either spontaneously generated or induced through transposon mutagenesis. Finally, the future development of high-throughput approaches based on whole-genome sequencing and comparative genomics will alleviate receptor discovery for an ever-growing number of newly identified bacteriocins (Sharp et al., 2017).

## Identifying Translocon Components Downstream of the Receptor

After binding to a specific receptor, bacteriocins translocate across the OM. The receptor itself can serve as a translocation pore, as for the group B pyocin S2 (White et al., 2017), or another protein can be recruited to the complex to serve as a translocator, as for the group A colicin ColE9 (Housden et al., 2013). Before establishing a translocation model, it is necessary to find all OM and periplasmic components of a bacteriocin’s translocon.

*Bacteriocin insensitive mutants* can be used to find translocon components other than the receptor. Mutant library screens have been particularly useful in this regard. For example, a screen of the Keio collection library for mutants insensitive but still capable of binding colicin S4 showed that *ompF*, *tolA*, *tolB*, *tolQ*, and *tolR* genes are all linked to its translocation. In the same study, it was found that after binding to the receptor OmpW, colicin S4 recruits OmpF and the Tol–Pal system to translocate across the OM (Arnold et al., 2009). A complication of this approach, however, is that *tol* gene knock-outs have altered membrane stability and permeability; deletion of *tolA* in *E. coli* leads to a pleiotropic phenotype characterized by outer membrane blebbing, release of the periplasmic content, increased sensitivity to cholic acid and SDS, and defective *O*-antigen polymerization (Germon et al., 2001). Indeed, *P. aeruginosa tolQRA* knock-outs are lethal (Wei et al., 2009). Therefore, using *tol* deletion mutants to check if bacteriocin entry is Tol–Pal dependent is not always feasible.

*Chimeric bacteriocins* have been effectively used to separate the receptor binding and translocation phases of the import process, which can be important for finding translocon components downstream of the receptor. This approach was successfully used to show that colicin Ia uses two copies of the Cir protein for OM translocation – one copy is used as the receptor and the other copy is used for translocation. A chimeric colicin Ia, in which the receptor-binding domain was replaced by that from colicin E3, required BtuB, the colicin E3 receptor, but also a copy of Cir and TonB for its killing activity. This experiment gave indications that one copy of Cir interacts with the receptor-binding domain of colicin Ia to concentrate the protein on the cell surface, while the other copy of Cir interacts with the translocation domain of this colicin so it can pass through the OM and enter the periplasm (Buchanan et al., 2007; Jakes and Finkelstein, 2010). However, direct binding of the Cir translocator with colicin Ia has yet to be demonstrated biochemically. *Receptor bypass* experiments have also been used to find translocon components other than the receptor. In such experiments, the OM is first permeabilized, usually by osmotic shock, so the receptor binding step is bypassed (Thomas and Valvano, 1993). This can be useful when a receptor for a bacteriocin under investigation is not known or when suitable chimeras are not available.

Very little is known about *inner membrane translocation* of Gram-negative bacteriocins. However, mutational analysis successfully identified some IM proteins necessary for transport of nuclease colicins. Walker et al. (2007) used a *ΔftsH E. coli* strain to show that the toxicity of all nuclease colicins (regardless of their Tol–Pal/Ton dependence) is dependent on FtsH, an inner membrane AAA+ ATPase/protease. This protease cleaves off the DNase domain during import to the cytoplasm (Chauleau et al., 2011; Mora and de Zamaroczy, 2014). In other studies, whole-genome sequencing of an *E. coli* mutant that is insensitive to colicin D gave indications that an inner membrane peptidase LepB is required for cell entry (de Zamaroczy et al., 2001). LepB is a key membrane component of the cellular secretion machinery, which releases secreted proteins into the periplasm by cleaving the inner membrane-bound leader. It was further shown that this protein binds to colicin D and probably directs it to the FtsH protease for cell entry (Mora et al., 2015).

Since IM translocation components are a part of protein translocation systems that are well conserved across different Gram-negative bacteria, it is possible to study the import of bacteriocins from other species using *E. coli* as a model system. The only limitation here is to bypass all the species-specific translocation steps that are mostly localized to the outer membrane. Hence, Mora et al. (2015) used a colicin D/klebicin D hybrid in which the N-terminal import domain of klebicin D was replaced with that of colicin D. Klebicin D targets *Klebsiella* species, but when fused to the receptor binding and the translocation domain of colicin D it can also kill *E. coli* obviating the need for a *Klebsiella* knock-out library. In this way, it was found that klebicin D, like colicin D, uses LepB for import (Mora et al., 2015).

## Deconstructing the Protein–Protein Interactions of Bacteriocin Translocons

Several approaches can be taken to define binding epitopes and binding induced conformational changes within bacteriocin translocons. PPIs between translocon components have to be first confirmed *in vitro* and *in vivo*. A common approach is *gene complementation* using a bacteriocin-resistant mutant or a bacteriocin-resistant species (Kjos et al., 2014), where the establishment of bacteriocin sensitivity can confirm the involvement of components in bacteriocin import (Figure 2). Apart from gene complementation assays, interactions between components can be assessed *in vivo* by *pull-down* experiments. A way to “freeze” the translocon in the assembly phase is by *in vivo* cross-linking (Masi et al., 2007; White et al., 2017) or by a disulphide locked bacteriocin (Housden et al., 2005, 2013). In both cases, a bacteriocin must be able to bind to its receptor and trigger translocon assembly without fully traversing the cell envelope and killing the target cell. Bacteriocin-bound protein complexes can further be extracted from the outer membrane by affinity chromatography using a bacteriocin or its immunity protein as bait. Pulldowns followed by limited proteolysis of the complex, and mass spectrometry of recovered protein fragments, can indicate which binding epitopes are involved in the translocon. For example, Housden et al. (2013) designed a disulphide lock that forms between the TolB-binding epitope of ColE9 and periplasmic TolB following recruitment of OmpF in the OM. A histidine tag on TolB enabled a heptameric assembly of the ColE9–Im9 complex, BtuB, OmpF trimer, and TolB to be purified and analyzed by mass spectrometry. Finally, limited proteolysis, in combination with planar lipid bilayer experiments and native mass spectrometry, demonstrated that within the translocon, ColE9’s unstructured N-terminal region passes twice through its bound porin thereby presenting its TolB-binding epitope in a conformationally constrained orientation in the periplasm (Housden et al., 2013).

*Calorimetric measurements* have also been used extensively in colicin import studies (Housden and Kleanthous, 2011), in particular ITC. ITC parameters can provide evidence of conformational changes within the translocon (Housden et al., 2005). ITC has also been used to determine how a bacteriocin affects existing PPIs within the cell envelope. Changes in the heats of binding in presence of a bacteriocin can indicate if it abolishes or induces a certain PPI. In this way, it has been shown that ColE9 interacts with TolB when entering the periplasm, disrupting the TolB–Pal complex and stimulating formation of a TolA–TolB complex that traverses the periplasm (Bonsor et al., 2009). ITC combined with site-directed mutagenesis can map translocon-binding sites, as in the case of the ColE9 TolB-binding region. The favorable enthalpy and unfavorable entropy changes associated with ColE9-binding TolB correspond to a disorder-to-order transition that occurs when the intrinsically unstructured region of ColE9 folds and binds TolB (Loftus et al., 2006). In other words, ITC parameters can indicate not only which regions of bacteriocins and their translocon components interact, but also which regions undergo conformational changes and in which phases of the translocation process these conformational changes occur.

*Stopped-flow FRET* experiments demonstrated how a bacteriocin can remodel PPIs within the cell envelope during import. Papadakos et al. (2012a) used a series of pre-steady-state kinetic experiments utilizing FRET pairs of ColE9 TolB-binding epitope, TolB and Pal, to establish the kinetic basis for competitive recruitment of TolB by ColE9 during which Pal gets displaced from its TolB–Pal complex. Interactions between translocon components have also been investigated using *planar lipid bilayers*. For example, it has been shown that ColE1 occludes TolC channels and that ColE9 occludes OmpF channels in planar lipid bilayers, confirming previous findings that these OMPs are involved in bacteriocin translocation (Zakharov et al., 2004). This approach was also used to identify a TolC box in ColE1 (Zakharov et al., 2016).

## Assembling the Translocon

A major challenge in understanding protein bacteriocin import is to assemble their translocons for structural studies where, for the most part, only general import mechanisms have been described (Cascales et al., 2007; Ghequire and De Mot, 2014; McCaughey et al., 2016; White et al., 2017).

Since Gram-negative bacteriocins are large multi-domain proteins, it is thought they must unfold, either partially or completely, in order to translocate across the outer membrane. Introducing *disulphide bonds* to prevent conformational changes in certain regions of a bacteriocin has been a useful approach in delineating such structural changes (Penfold et al., 2004). Similarly, *protease cleavage sites* have been used to probe the accessibility of bacteriocin sequences within a translocon. Zhang et al. (2008) introduced unique EK cleavage sites in a disulphide-locked ColE9 at a number of locations to study the surface accessibility of colicin subdomains shortly after receptor binding. In this experiment, a disulphide-lock within the colicin was used to synchronize translocon assembly; disulfide bond reduction simultaneously triggers initiation of translocation in all bacterial cells. This enabled determination of EK cleavage site accessibility for different regions of the colicin, which gave important insight into the position of ColE9 in the assembled translocon (Zhang et al., 2008).

Studies are beginning to unravel the molecular mechanism(s) by which bacteriocins translocate across the OM. White et al. (2017) developed an *in vivo cross-linking strategy* to map the import of the pyocin S2 NTD through the FpvAI receptor (Figure 3). This approach depended on first blocking import of the NTD using a C-terminal GFP. GFP is able to withstand ∼200 pN of force whereas the PMF can only deliver ∼20 pN (Saeger et al., 2012). This strategy allowed the accumulation of translocation intermediates that would otherwise be undetectable by crosslinking. Variants of this GFP fusion were then generated in which benzoylphenylalanine was incorporated at different positions of the NTD using amber suppression and crosslinked following transport into *P. aeruginosa* cells. Detailed mass spectrometric analysis of crosslinked peptides demonstrated that the pyocin not only translocates through FpvAI but that it does so by a process which likely mimics that used by pyoverdine, the natural ligand for FpvAI (White et al., 2017).

An important aspect of nuclease bacteriocin import is the release of the tightly bound immunity protein. All nuclease bacteriocins are produced bound tightly (*K*_d_ ∼ 10^-14^ M at pH 7 and 25°C) to their immunity protein Papadakos et al. (2012b). The half-life for dissociation for this complex is several days yet killing occurs within minutes. Hence, it has been postulated that an energy transduction path exists that jettisons the immunity protein at the cell surface during import. Zhang et al. (2008) developed a sensitive *fluorescence assay* to investigate immunity release. The assay was based on release of a fluorescently labeled immunity protein into the cell supernatant. Bacteriocin import was synchronized using a disulfide-lock. In this way, fluorescently labeled Im9 was detected in the cell supernatant after the addition of a reducing agent and was dependent on the PMF across the inner membrane (Zhang et al., 2008; Vankemmelbeke et al., 2012), shown subsequently to be linked to global conformational changes within the colicin (Vankemmelbeke et al., 2013). How force might cause immunity dissociation has been investigated by *single molecule atomic force spectroscopy* (Farrance et al., 2013). These studies demonstrated that the ColE9 DNase–Im9 complex is exquisitely sensitive to mechanical deformation at the N-terminus of the nuclease, which could represent pulling into a cell, causing rapid dissociation of the immunity protein.

## Structural Biology Approaches for Studying Bacteriocin Import

Structural studies are important in gaining a molecular understanding of the bacteriocin translocation process. To-date, there are only a few structures of intact Gram-negative bacteriocins (Wiener et al., 1997; Soelaiman et al., 2001; Helbig et al., 2011; Klein et al., 2016). More informative, in terms of translocation mechanism, are structures of bacteriocins or bacteriocin domains bound to their receptor or a component of the translocon assembly (Kurisu et al., 2003; Loftus et al., 2006; Buchanan et al., 2007; Sharma et al., 2007; Zhang et al., 2009; Housden et al., 2010; White et al., 2017). These structures give information about conformational changes that a bacteriocin induces and shed light on the import mechanism.

Crystallization of bacteriocins can often be challenging due to intrinsically disordered and flexible regions in these proteins. Flexibility can be reduced by deletion of these sequences or by the introduction of intramolecular disulfide bonds (Klein et al., 2016). To complete a structure of a bacteriocin it is often necessary to substitute missing X-ray diffraction data with results from other experiments. Modeling of data from *analytical ultracentrifugation* (Manon and Ebel, 2010) and *NMR experiments* (Collins et al., 2002; Hecht et al., 2009) can help in filling-in missing parts of a bacteriocin structure. Results from *SAXS* experiments can also be combined with diffraction data; a bacteriocin can be treated as a flexible system, namely by the ensemble optimization method, which enables a distribution of conformations to be included in the final model (Johnson et al., 2017).

Structures of *bacteriocin–receptor and bacteriocin–translocator complexes* are essential to understand bacteriocin import mechanisms. When compared to receptor–cognate ligand complexes, these structures can show to what extent a bacteriocin mimics the ligand (if at all) when traversing the cell envelope. For example, co-crystal structures for E-type colicins bound to the BtuB receptor (ColE2, ColE3) show that the colicins do not mimic the interactions of the ligand and do not induce conformational changes within the globular N-terminal plug domain of BtuB indicative of transport. This contrasts the situation of pyocin S2-NTD bound to its receptor FpvAI where the structure clearly supports a model (validated by crosslinking data) that the pyocin mimics the endogenous ligand, pyoverdine, and transports through the receptor in a TonB-dependent manner (White et al., 2017). For the E colicins (all of which require the Tol complex), entry to the periplasm requires OmpF or OmpC. These porins acts as translocators, which is supported by structures of OmpF in which fragments of E colicins are bound within its lumen (Yamashita et al., 2008; Housden et al., 2010) and by planar lipid bilayer experiments where colicin fragments block ion conductance (Zakharov et al., 2004).

The development of structure–prediction algorithms could broaden the understanding of bacteriocin translocons (Delarue and Koehl, 2018). For instance, protein fold predictions have already been employed in structure determination of a group A colicin translocon component, TolQ (Ovchinnikov et al., 2015). Additionally, future developments in translocon structures will undoubtedly involve cryo-EM methods. It may even be possible to eventually capture a bacteriocin in-transit and use cryo-tomography to map out its interactions within the cell envelope.

## Viewing the Import Process in the Context of Outer Membrane Organization

**FIGURE 4:**
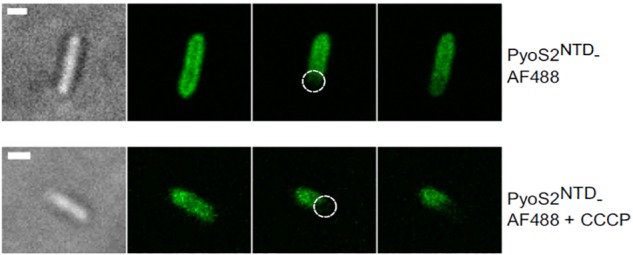
FRAP experiments can be used to show that bacteriocin import is a PMF-dependent process. *P. aeruginosa* PAO1 cells are labeled with pyocin S2 AF488. The bleached region is highlighted (dashed circle). FRAP suggests pyoS2NTD-AF488 has translocated to the periplasm, where it can diffuse laterally. Absence of FRAP observed when cells are treated with 100 μM CCCP indicates that the pyocin remains bound to FpvAI in the OM and that the PMF is necessary for pyocin translocation. Scale bars, 1 μm [image taken and used with permission from White et al. (2017), CC BY-NC-ND 4.0].

**FIGURE 5:**
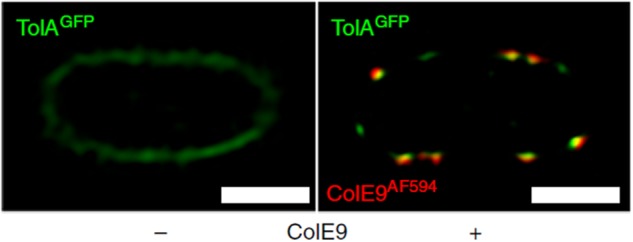
Fluorescently labeled bacteriocins can be used to track outer and inner membrane protein clusters; used by Rassam et al. (2018) to show that bacteriocin-induced clustering of TolA in the IM mirrors that of OMPs in the OM. 2D-SIM z-slice showing significant co-clustering (yellow fluorescence) of GFP-TolA and ColE9AF594 in the IM and OM, respectively. Scale bars, 1 μm [image taken and used with permission from Rassam et al. (2018), CC BY-NC-ND 4.0].

Bacteriocins have become valuable tools with which to investigate spatiotemporal organization in the cell envelope by fluorescence microscopy (Kleanthous et al., 2015). Fluorescently labeled bacteriocins have been used in conjunction with *single particle tracking TIRF microscopy* to show that OMPs display highly restricted mobility in the outer membrane due to the formation of supramolecular assemblies called OMP islands, which also explains the lack of fluorescence recovery for labeled OMPs in FRAP experiments (Figure 4). TIRF microscopy of bacteriocin-labeled OMPs has also shown that OMP islands move to the poles as new islands appear in the membrane (Rassam et al., 2015). Even more remarkably, the restricted mobility that is characteristic of OMPs within OMP islands becomes imprinted on inner membrane proteins when the bacteriocin forms its translocon across the two membranes (Figure 5; Rassam et al., 2018).

*Fluorescently labeled bacteriocins* have also been used for dissecting their import mechanism. GFP was deployed by White et al. (2017) to visualize association of pyocin S2 with *P. aeruginosa* cells, block translocation, and trap the pyocin within its receptor, FpvAI, for subsequent crosslinking studies. By switching to an organic dye (AF488), White et al. (2017) demonstrated import of the pyocin S2 domain since in contrast to GFP-labeled protein, fluorescence recovery was observed for AF488-pyocin S2-labeled cells in FRAP experiments. Imported fluorescent protein was also protected against exogenously added protease.

*Live cell imaging* can also be used to dissect the directionality of PPIs formed between bacteriocins and their translocators. Housden et al. (2018) used a combination of molecular dynamics simulations, fluorescence microscopy, and single channel recording planar lipid bilayer measurements to unambiguously demonstrate from which side of the OM different OBSs of ColE9 associated with the lumen of the porin. The intrinsically unstructured N-terminal region of ColE9 houses two OBSs (OBS1 and OBS2) that reside within the pores of OmpF and that flank an epitope that binds periplasmic TolB. The studies revealed that OBS2 binds OmpF from the extracellular side, while the interaction of OBS1 occurs from the periplasmic face of OmpF, which ensures constrained presentation of the TolB epitope within the bacterial periplasm (Housden et al., 2018).

## The Energetics and Kinetics of Bacteriocin Import

The energetics of bacteriocin translocation are still controversial. Indeed, little is known about the energy dependence of individual translocation steps. Since bacteriocins are folded proteins, import is likely to rely on the input of energy, the main source being the PMF generated across the IM. However, some studies suggest that OM translocation is energy independent, as in the case of colicin A (Bourdineaud et al., 1990). Nevertheless, live cell imaging studies are beginning to show the link between PMF, Ton, Tol, and bacteriocin import (White et al., 2017; Rassam et al., 2018).

A way of testing if energy is necessary for a certain phase of bacteriocin import is to disrupt the PMF and then assess import with a suitable assay. Protonic ionophores have been used for this; for example, CCCP was used in FRAP experiments to show that PMF is necessary for import of pyocin S2 (Figure 4; White et al., 2017). Disulphide locked colicins and fluorescently labeled immunity proteins have been used to study the energy dependence of immunity protein release (Vankemmelbeke et al., 2012). A sensitive assay for detecting immunity protein release (described above) was used to confirm that both TolB and TolA are necessary for this process in ColE9 import and to define which regions of TolA are engaged. TolA is anchored in the cytoplasmic membrane via a single transmembrane region that interacts to TolQ and TolR and drives a TolA conformational change in response to PMF (Germon et al., 2001). Vankemmelbeke et al. (2009) focused on residue H22 of the TolA IM region that has been previously linked to energy-dependent conformational change of this protein (Larsen et al., 2007). They showed that an alanine mutation of this residue disrupts immunity protein release, which indicated that an energy-dependent conformational change of TolA is essential for nuclease colicin Im9 release at the cell surface. This points to a role for TolA in transducing cellular energy in a manner similar to that described for TonB (Postle and Larsen, 2007), but the mechanism remains unknown. Bonsor et al. (2009) went on to show that it is the contact between TolA and TolB within the periplasm that is the necessary energy-transduction step for nuclease colicin Im release. One model proposes that the rotation of Tol complex transmembrane helices is transduced into a conformational change in TolA that affects its interaction with the TolB–bacteriocin complex and triggers Im protein dissociation (Papadakos et al., 2012). New methodological approaches will be necessary to dissect how the electrochemical potential of the PMF is transduced by the Tol and Ton systems into the mechanical action of bacteriocin import.

Little is known of the kinetics of bacteriocin import beyond the onset of cellular changes these protein toxins induce. Early studies showed that conformational flexibility is a prominent bacteriocin trait, determining the kinetics of import (Duche et al., 1994). Additionally, bacteriocin charge (Walker et al., 2007) and cell envelope properties related to lipid composition are factors that influence the speed of import (Bourdineaud et al., 1990; Walker et al., 2007). In the case of pore forming colicins, measuring cytoplasmic potassium efflux caused by colicin A inner membrane insertion was used to show how inner membrane fluidity influences the kinetics of translocation (Bourdineaud et al., 1990). In the case of nuclease colicins, Walker et al. (2007) observed that inner membrane charge impacts the rate of import. In this study, a strain of *E. coli* in which the level of anionic phospholipids was regulated by isopropyl β-D-thiogalactopyranoside induction was used to show that both inner membrane and bacteriocin charge influence the rate of cell entry. Import kinetics were measured indirectly, through the efficiency of cell killing or DNA damage. Still, little is known about the kinetics of individual translocation steps and how PPIs between translocon components influence the rate of import. Therefore, to understand import kinetics in more detail, methods for direct measurement of individual translocation steps will have to be developed, most likely based on single molecule methods.

## Future Perspectives

As protein bacteriocins begin to be exploited as therapeutics for multidrug-resistant bacteria so our ability to understand their import mechanisms needs to develop (Behrens et al., 2017). Various approaches, ranging from classical biochemical methods to advanced molecular genetics, have been used over the years for translocon component discovery. The development of high-throughput approaches based on whole-genome sequencing and comparative genomics can speed up this process in the future. Calorimetric measurements have been used extensively to identify binding epitopes and to study the nature of interactions between translocon components. Moreover, implementation of various microscopy techniques, channel conductivity measurements, *in vivo* cross-linking, and disulphide locking of bacteriocins have been exploited for determining the directionality of PPIs within the translocon and for identifying entry paths that bacteriocins use when crossing the OM. Finally, *in vivo* imaging of cells bound to fluorescently labeled bacteriocins has enabled visualization of the import process for the first time at single cell level and provided new tools for probing the spatiotemporal organization of the cell envelope.

While substantial knowledge about OM translocation of bacteriocins has been gained over recent years, the IM component of translocation remains to be elucidated. Many questions remain unanswered – which proteins are involved in bacteriocin IM translocation, how is this process energized, which parts of a bacteriocin are necessary for IM translocation, and which parts enter the cytoplasm. Additionally, a clear link between the OM and IM components of bacteriocin translocons still has to be revealed. For example, are nuclease bacteriocins brought into the periplasm in their entirety before import across the cytoplasmic membrane begins? Finally, the study of bacteriocins in the context of cell envelope organization has breathed new life into bacteriocin research.

## Author Contributions

IA and CK contributed to the original manuscript and the editorial changes.

## Conflict of Interest Statement

The authors declare that the research was conducted in the absence of any commercial or financial relationships that could be construed as a potential conflict of interest.

## Abbreviations

CCCP: carbonyl cyanide 3-chlorophenylhydrazone
ColE9: colicin E9
CPA: common polysaccharide antigen
EK: enterokinase
FRAP: Forster recovery after photobleaching
FRET: Forster resonance energy transfer
Im9: ColE9 immunity protein
IM: inner membrane
ITC: isothermal titration calorimetry
LPS: lipopolysaccharide
MMBL: monocot mannose-binding lectin
NMR: nuclear magnetic resonance
NTD: N-terminal domain
OBS: OmpF-binding site
OM: outer membrane
OMP: outer membrane protein
PMF: proton motive force
PPI: protein–protein interactions
SAXS: small-angle X-ray scattering
TIRF: total internal reflection fluorescence
